# Personal

**Published:** 1899-05

**Authors:** 


### PERSONAL.

Dr. Charles Jewett, of Brooklyn, professor of obstetrics and pediatrics at the Long Island College Hospital, was, on April 29, 1899, appointed by the trustees to be president of the faculty of that institution—a place made vacant by the resignation of Dr. Alexander J. C. Skene. Dr. Jewett has been a prominent teacher for nearly twenty years and is an author of one of the best books on obstetrics that has been published in English. His election to the presidency of Long Island College Hospital is a well-deserved honor.

---

Dr. Thaddeus A. Reamy, of Cincinnati, was tendered a complimentary dinner, on Friday, April 28, 1899, in celebration of his 70th birthday. It was attended by 170 of his professional friends, with here and there a layman of conspicuous prominence. Among those from nearby cities were Dr. Joseph McDowell Mathews, president of the American Medical Association, and Dr. Lewis Samuel McMurtry, of Louisville; and Dr. David Barrow, of Lexington. Dr. McMurtry responded to a toast in his usual felicitous manner, the sentiment assigned him being, A pioneer of gynecology.

Dr. Reamy has been a conspicuous character in the drama of medicine for these many years and it must have been one of the proudest moments of his life when he received such a token of respect and affection by 200 of his fellow-citizens and colleagues.

---

Dr. Wilhelm Gaertner, of Buffalo, has removed his office from 554 Michigan street to 602 Ellicott street, corner of Goodell street. Hours: 8 to 9 a. m., 1 to 2.30 and 6 p. m. Branch office, 244 Goodell street, corner of Cherry and Hickory streets. Hours: 9 to 10 a. m., 2.30 to 4 and 7.30 p. m.

---

Dr. Marshall Clinton, of Buffalo, formerly assistant surgeon of the 202d Regiment N. Y. V. Infantry, was recently mustered out of the United States' service and has returned to Buffalo. He has resumed the practice of his profession and his office is at the corner of Allen and North Pearl streets.

---

Dr. Albert H. Macbeth, of Cambridge Springs, Pa., formerly of Buffalo, sailed for Europe, March 25, 1899, on the Cunard liner Campania, to be absent about five months. It is possible Dr. Macbeth may visit the Philippine islands during his absence.

---

Dr. Burt C. Johnson, of Buffalo, announces that on May 2, 1899, he will remove his office and residence from 274 E. Utica street, to 1404 Main street, near Utica. Hours: 8 to 9 a. m., 1 to 3 and 7 p. m. Telephone Bryant 164 D.

---

Dr. B. H. Putnam, of North East, Pa., has been appointed surgeon of the Lake Shore & Michigan Southern Railway Company, vice Dr. D. D. Loop, resigned. The appointment took effect April 1, 1899.

---

Dr. Charles A. Wall, of Buffalo, sailed for Europe by the American line, April 12, 1899, for an absence of four months. He was accompanied by Mrs. Wall and children.

---

Dr. William House, of Buffalo, has removed from 1106 Main street to 26 Allen street.

DR. J. HENRY DOWD, of Buffalo, has removed his office and residence from number 223 to 288 Franklin street. Hours: 9 to 1. Sundays, 2-4. Telephone.

DR. WILLIAM G. BISSELL, of Buffalo, has removed his office and residence from the Buckingham to 143 Elmwood avenue. Hours: 12-2 and 6-8 p. m.

DR. EDWARD J. MEYER, of Buffalo, took passage for Europe by the North German Lloyd line April 19, 1899. He was accompanied by Mrs. Meyer.

